# Identifying behaviour change techniques, technical features and implementation options for a virtual reality intervention to motivate adult smokers to quit: A focus group study with healthcare and virtual reality experts

**DOI:** 10.1177/20552076251330510

**Published:** 2025-03-28

**Authors:** Tosan Okpako, Corinna Leppin, Alessandro Chincotta, Dimitra Kale, Olga Perski, Jamie Brown

**Affiliations:** 1 Department of Behavioural Science and Health, 4919 University College London, London, UK; 2 Herbert Wertheim School of Public Health and Human Longevity Science, 8784 University of California, San Diego, La Jolla, CA, USA; 3 Faculty of Social Sciences, 7840 Tampere University, Tampere, Finland; 4 Spectrum Research Consortium, Edinburgh, UK

**Keywords:** Virtual reality, behaviour change, digital health, ontologies, person-based approach, eHealth, smoking, qualitative

## Abstract

**Objective:**

In this study, individuals working in healthcare or virtual reality (VR) were invited to contribute towards developing a VR intervention to encourage adults to quit smoking, by building upon user-generated ideas from a previous co-design study with adult smokers.

**Methods:**

Three online focus groups were with healthcare workers (*n* = 26), and one was with VR experts (*n* = 4). Transcripts were analysed using thematic analysis.

**Results:**

In the first theme, experts agreed that previous co-design content showing the outcomes of quitting or not quitting could be helpful. Suggested behaviour change techniques included ‘prompt comparative imagining of future outcomes’, and ‘imaginary reward’. Experts suggested a format where users could customise an avatar and select what content they see, creating a tailored narrative. There was a subtheme about the potential unsuitability of co-design content based on extreme fear appeals, which experts thought could trigger fatalism or defensiveness. The second theme covers considerations to make VR safe and inclusive. For example, making the experience seated for those with limited mobility, hygiene protocols and a screening process to exclude smokers at risk of harm (individuals with frailty, photosensitive epilepsy or a serious mental health condition). The last theme outlines the benefits and potential barriers of implementing VR with the ‘ask, advise, act’ model for smoking cessation used in healthcare contexts.

**Conclusion:**

Findings suggest that VR has the potential to deliver embodied, interactive and customisable smoking cessation messages, rooted in behavioural theory. The suggestions will inform the development of a prototype which will subsequently be evaluated.

## Introduction

Motivation to stop smoking is a key predictor of quit attempts.^1^ However, survey data indicates that the majority of the English smoking population do not have plans to quit within the next 30 days.^2^ This suggests that more work should be done to encourage quitting in adults who smoke (herein referred to as smokers) to meet the government target of a smoke-free England by 2030.^3^ Interventions that encourage and assist cessation are among the most cost-effective and lifesaving in high-income countries.^4^ This study concerns the development of a new virtual reality (VR) intervention aimed at prompting quit attempts in adult smokers.

Mass media campaigns, print materials and cigarette package warnings are effective population-level interventions aimed at increasing motivation to quit.^4,5^ However, the individual-level approach towards encouraging smoking cessation with the highest potential reach is opportunistic advice from a healthcare worker.^4^ UK guidelines recommend that patients visiting their general practitioner (GP) should be annually assessed for smoking, followed by brief behavioural or pharmaceutical support.^6^ This increases the average 6–12 months abstinence rate by two percentage points.^4^ However, survey data from adult smokers in England indicated that less than a third received advice to quit from their GP in the past year.^7^ Additionally, among smokers who made a quit attempt between 2018 and 2023, the proportion who were motivated by a health professional's advice fell from approximately 14.2% to 8.5%.^8^ Therefore, developing new and convincing interventions to motivate smokers to quit could reduce smoking prevalence.

Novel interventions based on VR have been used to support healthy behaviour in areas such as physical activity, alcohol use and diet.^9^ In a systematic narrative review of VR interventions for health behaviour change, 84% of evaluations (randomised control trials and non-randomised studies) reported positive results.^9^ A few pilot studies have used VR to encourage smoking cessation. For example, in a sample of Italian young adults, a VR video showing a hotel room fill up with cigarettes before transforming into a hospital room resulted in a greater increase in motivation to quit compared to traditional cigarette package warnings.^10^ Conversely, health warnings about hookah presented to university students through VR resulted in smaller shifts in attitudes and beliefs compared to traditional online warnings.^11^ More work is needed to investigate what types of messages are most effective in triggering behaviour change in smokers and how to best use VR's technological potential.

VR involves wearing a headset, which provides a 360-degree view of a digital world.^12,13^ VR interventions include both its technical features and content.^14^ Content can refer to the story narrative. Behavioural theory and specific behaviour change techniques (BCTs) can be incorporated into narratives.^15,16^ BCTs represent the smallest components of an intervention that have the potential to trigger behaviour change (active ingredients).^17^ Specifying BCTs when developing an intervention supports descriptions of the causal pathway (mechanism of change) and evaluation.^17^ Additionally, the technical features of VR refer to the characteristics and delivery features of the technology (graphics, controllers, audio, etc.) and how they increase or disturb the cognitive illusion of being part of the virtual world (presence).^14,18,19^

A key benefit of delivering smoking cessation interventions via VR is that the small size and weight of the equipment and low baseline skills and expertise needed for setup make it possible to implement in varied settings, such as people's own homes, GP offices, or in community centres.^20^ For example, smoking cessation VR has been successfully piloted in a dental setting.^21^ Although VR-based interventions could usefully be delivered in people's homes, ownership of headsets is currently low in the UK, at approximately 4% in 2021.^22^ There is potential for increased exposure to VR-based interventions if delivered in diverse settings with high footfall. Furthermore, if delivered in a healthcare setting, adding human support is known to improve engagement with digital interventions.^23^ However, few studies have explored the benefits, drawbacks and organisational readiness to use smoking cessation VR interventions in different settings.^20^

This study is nested within a larger project aiming to develop and evaluate a VR scenario that encourages adults to quit smoking. In line with the person-based approach (PBA), stakeholders (those with personal or professional interests that coincide with the intervention) were invited to actively contribute to the intervention's development.^24,25^ First, the views of adult smokers were gathered during a previous focus group study, which focused primarily on the scenario's narrative content. These suggestions focused either on the use of graphic fear appeals or emotive content showing the future outcomes of quitting or not quitting.^26^ However, the outcomes of co-design sessions with future end-users are usually not service-ready solutions but user-generated ideas.^27^ Therefore, this second study builds upon these ideas, involving experts working in healthcare or VR.

The creation of health VR applications is at the centre of multiple sectors such as 3D art, computer science, healthcare practice and behavioural research.^20^ Therefore, this present study aimed to elicit knowledge and ideas about implementation approaches, BCTs and technical features to incorporate into the user-generated ideas collected from adult smokers from a previous study. In particular, this study sought to explore what BCTs healthcare experts working in evidence-based smoking cessation recommend for a VR smoking cessation intervention. We also aimed to investigate which technical features would better immerse adult smokers within the VR intervention, while being safe, feasible and applicable across different delivery platforms. Finally, this study explored which settings a smoking cessation VR intervention could usefully be implemented in.

## Methods

### Study design

This is a qualitative study, using focus groups. In contrast to one-to-one interviews, we chose to use focus groups because the group dynamic can encourage spontaneity in the expression of views and facilitate collaborative efforts among experts.^28^ A protocol was pre-registered on the open science framework (OSF) (https://osf.io/2fxjh/). This study is reported in line with the Consolidated Criteria for Reporting Qualitative Studies (COREQ) checklist.^29^ The complete COREQ checklist is available in Supplemental Material 1.

### Participant selection

#### Inclusion criteria

Eligible experts had to speak English and have research or work experience in healthcare, smoking cessation, computer science or VR.

#### Sampling strategy

We recruited experts using snowball techniques. We emailed existing contacts to share the recruitment poster with their networks and place on mailing lists. The poster was also shared on LinkedIn.com. To assess eligibility, we used a secure online REDCap survey^30^ to collect data on preferred attendance dates, age, gender, ethnicity, and professional background (Supplemental Material 2). As stakeholders in healthcare are frequently not included in the development of VR health applications in a substantive way, this study used purposive sampling to obtain a variety of health professionals.^20^

The focus groups were segmented by professional background. Intersectoral collaboration is useful as it can generate new understandings that cross disciplinary boundaries and mitigate cognitive biases.^23,27^ However, different sectors have different sets of cultures, practices and assumptions.^31^ One session is unlikely to be enough time to produce a shared language for productive discussion. We emailed eligible experts to schedule and confirm attendance. All experts who confirmed their attendance via email beforehand showed up to the focus groups.

### Setting

The focus groups were conducted online via video conferencing (Microsoft Teams) and were recorded. TO moderated the discussion, while CL and/or AC took observational notes. All participants were required to keep their cameras on. We chose online focus groups to eliminate geographical limitations and for greater scheduling flexibility. Previous comparisons indicate the data quality in online focus groups can be comparable to those conducted face-to-face.^32^

### Data collection

Each focus group lasted approximately 60 min (mean = 60.5, standard deviation = 5.5). There were separate question routes for each set of experts (Supplemental Material 3).^33^ Both question routes began with broad questions about VR or smoking cessation. Experts were then advised that the goal was to refine and finalise user-generated ideas from a previous study with adult smokers. For that section of the focus group, we presented a slideshow summary of these ideas for the experts to provide comments. Copies of the slide presentations are available on OSF https://osf.io/2fxjh/. A full description of the user-generated ideas is published elsewhere.^26^

To summarise, adult smokers suggested two broad categories of content. The first type of content was ‘coming of age’ stories set in the future showing either the benefits of quitting or the consequences of not quitting, triggering hope or regret. Family members were identified as a key influence on smoking behaviour, so were drawn as supporting characters in the suggested content. The second content category was ‘horror’ stories, which focused on extreme fear appeals and surreal imagery. The goal was to trigger anxiety and fear around smoking. Preliminary feedback on these user-generated ideas highlighted concerns about using extreme fear appeals. Therefore, we included a specific prompt about this when asking experts about the horror content. However, the moderator only used this prompt if experts expressed an unfavourable opinion during the focus group, which occurred in the first three groups.

### Analysis

We used a variant of reflexive thematic analysis (TA) described by Braun and Clarke as ‘codebook TA’. We chose this method of analysis as it allows for a structured approach to coding, while still acknowledging that the data is contextually situated and the active role of the researcher in knowledge production (reflexivity).^34^ TA was conducted in six iterative steps: (1) familiarisation with the data, (2) generating initial codes, (3) searching for themes, (4) reviewing themes, (5) defining and naming themes and (6) producing the report.^35^

The coding was a mixture of inductive and deductive, based on the taxonomy developed by Motejlek and colleagues for VR development and the behaviour change ontology.^17,19,36^ The use of these taxonomies provided a lens to make sense of the data, rather than forming the basis of a hypotheticodeductive model.^34^ The development of themes was inductive and captured codes across these taxonomies, while also including inductive codes that fell outside the scope of each. TO was the primary data analyst and the whole research team (TO, CL, AC, JB, DK, OP) discussed and agreed upon the final themes. In producing this report, we supported themes with participant quotes giving their age, gender (male/ female) and profession. A high level-summary of results was sent to participants, who agreed that it accurately represented their discussions. Our reflexivity statement can be found in Supplemental Material 4.

### Changes from pre-registration

In the pre-registered protocol, we anticipated time and recruitment challenges so specified a target sample size of approximately 12 experts. However, the recruitment success of healthcare experts was greater than anticipated. Therefore, we expanded the sample size to 30 to obtain a greater number of viewpoints and improve theoretical saturation.

We initially analysed the transcripts using BCTTv1 (behaviour change technique taxonomy version 1). However, after the initial analysis, we re-analysed the data using the most current version of the BCTO (BCT ontology) and the style of delivery ontology.^17,36^ Both form a part of the wider behaviour change intervention ontology.^37^ Compared to the BCTTv1, the BTCO has greater completeness, more precise and clear groupings and links to other aspects of an intervention scenario such as the mechanisms of action ontology.^17^

### Ethical approval

UCL's Research Ethics Committee granted ethical approval (ID: 25627.002). All participants gave informed consent, and we removed identifying information from the transcripts.

## Results

### Participants

From March to June 2024, there were 30 participants, across four groups. The size of groups ranged from 4 to 9 experts. Table 1 shows the composition of each focus group and the total sample. The mean age of experts was 41 years old (standard deviation = 12), 70% of the sample were female and 70% were of white ethnic origin. The first three focus groups were for healthcare experts and the last focus group was for VR experts. While the majority of healthcare experts were stop-smoking advisors, the sample also included doctors, pharmacists and nurses with experience in smoking cessation. All VR experts included in this study had prior experience developing VR for health-related problems such as smoking cessation, physical rehabilitation and mental health. While we did not explicitly ask experts about their smoking status, one expert in the second focus group mentioned that they used to smoke.

**Table 1.** Sample description.

Demographic and professional characteristics	Smoking cessation and healthcare experts			VR experts	Total (*N* = 30)
	Group 1 (*n* = 8)	Group 2 (*n* = 9)	Group 3 (*n* = 9)	Group 4 (*n* = 4)	
Age mean (standard deviation)	42.4 (15.5)	48.4 (8.9)	38.8 (11.8)	31.0 (11.4)	**41.4** (**12.8)**
Female % n	50.0% (4)	77.8% (7)	88.8% (8)	50.0% (2)	**70.0** (**21)**
Any White background % n	75.0% (6)	66.7% (6)	44.4%(4)	25.0% (1)	**70.0** (**21)**
Profession % n					
*Tobacco dependency advisor/ stop smoking advisor*	50.0% (4)	55.6% (5)	0.0% (0)	-	**30.0%** (**9)**
*Medical doctor* ^a^	25.0% (2)	11.1% (1)	11.1% (1)		**13.3%** (**4)**
*Dentist*	0.0% (0)	0.0% (0)	11.1% (1)	-	**3.3%** (**1)**
*Pharmacist*	12.5% (1)	0.0% (0)	44.4% (4)	-	**16.7%** (**5)**
*Psychiatrist*	0.0% (0)	0.0% (0)	11.1% (1)	-	**3.3%** (**1)**
*Nurse/ student nurse*	0.0% (0)	22.2% (2)	0.0% (0)	-	**6.7%** (**2)**
*health care assistant*	0.0% (0)	11.1% (1)	0.0% (0)	-	**3.3%** (**1)**
*Other health professions* ^b^	12.5% (1)	0.0% (0)	22.2% (2)	-	**10.0%** (**3)**
* Computer science academia*	-	-	-	50.0% (2)	**6.7%** (**2)**
*Unity developer*	-	-	-	25.0% (1)	**3.3%** (**1)**
*Technical artist*	-	-	-	25.0% (1)	**3.3%** (**1)**

a Two medical doctors were hospital based, a third was primary care based (general practitioner).

b Other health professionals included a service manager, general practitioner's assistant, and healthy living hub advisor.

### Themes

#### Overview

We developed three main themes and one subtheme. The first theme was ‘Built upon VR content – “Choose your own adventure”’ and under this was the subtheme ‘Unsuitable VR content – The potential problems with extreme fear appeals’. The next theme was ‘Technical features – safe, inclusive and easy to use VR for a smoking population’ and the last was ‘Implementation – VR within the “ask, advise, act” model in healthcare settings’. Table 2 gives an overview of these themes and their related codes. The full codebook is available on the OSF https://osf.io/2fxjh/.

**Table 2.** Themes and related codes.

Theme	Related codes	
Theme 1: Built upon VR content- ‘Choose your own adventure’	Tailor interactions appropriately (Personalisation) Assess past history of quit attempts (Personalisation) Rewards-based gamification Personalisation – story decision tree Inform about negative health consequences BCT Inform about positive health consequences BCT Imagine reward BCT Prompt comparative imagining of future outcomes BCT	Re-attribute cause BCT Present information from credible influence BCT Inform about negative social consequences BCT Adopt changed self-identity BCT Affirm valued self-identity BCT Empathic communication style Non-judgmental communication style Intelligent agents (and the challenges of artificial intelligence) Modelling vs Cinematography Customisable avatars Audio features
Subtheme 1: Unsuitable VR content- The potential problems with extreme fear appeals	Ethicality of fear Scepticism regarding fear and surreal images	Surreal images entertaining Increase salience of consequences BCT
Theme 2: Technical features- safe, inclusive and easy to use VR for a smoking population	Cognitive load and physical fatigue Physical challenges with VR Psychological challenges with VR Cybersickness General controllers vs User tracking Hygiene concerns	Locations and safety Screening Severely unwell patients Tripping prevention Language translations Age and technology acceptance Ease of use Healthcare locations
Theme 3: Implementation- VR within the ‘Ask, advise, act’ model in healthcare settings	Occupational or commercial locations Healthcare locations Age and location Clinician resistance to giving brief advice Implementation- time and human resource restrictions	Efficiently identifying and reaching smokers Assess past history of quit attempts (Personalisation) Give options for additional and later support Encourage pharmacological support BCT

Theme 1 (Built upon VR content – ‘Choose your own adventure’) focuses on the suggested BCTs to incorporate into the user-generated ideas from a previous focus group with adult smokers. Experts agreed that showing the user the future could be effective. The main BCTs recommended were ‘prompt comparative imagining of future outcomes’, ‘imaginary reward’ and ‘information about positive health consequences’. This theme also highlights the technical delivery features that could make the VR content interactive and tailored. The consensus was to have a ‘choose your own adventure’ narrative, where the scenario each user receives will depend on their selected preferences or demographic profile. In the subtheme (Unsuitable content – The potential problems with extreme fear appeals) experts were not convinced by the user-generated content that focused on extreme fear appeals. They believed it could trigger fatalism or defensiveness. Some experts also thought that surreal images could trigger a humorous reaction.

The second theme (Technical features – safe, inclusive and easy to use VR for a smoking population) focused on technical considerations to make the VR safe and inclusive, especially if implemented in a healthcare context. Examples of recommendations include making the experience seated, limiting flashing images and limiting fast movements to prevent cybersickness. Experts also recommended a hygiene protocol (disinfecting the headset between uses) and a screening process to exclude smokers at risk of harm (those with a serious mental health condition, photosensitive epilepsy or physical frailty). The last theme (Implementation – VR within the ‘ask, advise, act’ model in healthcare settings) focuses on suitable locations for implementation. It is based around the ‘ask advise act’ (3As) model of smoking cessation support. The 3As model is derived from the 5As model (ask about smoking status, advise patients to quit smoking, assess readiness to quit, assist with making a quit attempt, and arrange a follow-up).^38,39^ Overwhelmingly, experts suggested healthcare locations. This theme explains the challenges and benefits of incorporating VR into the 3As model used by healthcare professionals, particularly, difficulties in the ‘ask’ phase, such as time constraints, and the need for follow-up support (‘act’) after the VR scenario.

#### Theme 1: built upon VR content – ‘choose your own adventure’

This theme explores the ways in which the ‘coming of age’ content, suggested in a previous co-design study with smokers,^26^ was refined by experts in this current study. Adult smokers previously suggested content set in the future, showing the outcomes of quitting or not quitting. Healthcare experts agreed that this type of content could be effective as it draws upon methods they have used in their practice. However, healthcare experts predominantly preferred showing positive content. We coded participant responses to match with BCTs such as ‘comparative imagining of future outcomes’, ‘imaginary reward’ and ‘inform about positive health consequences’. Near and distant rewards of quitting smoking that experts emphasised were health, quality of life, appearance and money-related.

*I think it's always really difficult to kind of imagine yourself in a scenario really far down the line. But I think it's always useful to kind of try and do that and visualise it, rather than just imagining it, to actually see it.* **(50F, stop smoking advisor)**

*I know last Stoptober, posters were based around, You won't smell like an ashtray. You can, get up the stairs without getting breathless. You can actually, have a few pennies spare at the end of the week* **(52M, clinical lead for tobacco dependency and mental health)**

However, there were some concerns that future-oriented content might not be effective for older smokers, who felt themselves to already be in the future.

*But people who are a bit older and smoke, they tend not to be too bothered about fear because they're already in the future in their minds. So, they think ‘I've already smoked for 60 years. You know, this hasn't happened to me. I don't see why it would’. So, it really depends on the target audience as well. If it's for younger people, yes, I think that probably has an impact on it*. **(27F, tobacco dependency advisor)**

*I find that people, some people, will say, ‘Oh, I've smoked since I was 15, so I'm not going to give up now’.* (**51F, health care assistant and student nurse)**

Smoking cessation experts also favoured using language and communication styles that were empathetic and non-judgemental. They believed that this would decrease defensive reactions.

*So, we sort of do it from a different approach. They think we're actually there to help them and not judge them* (**29M, tobacco dependency advisor)**

Additionally, between and within focus groups there was some debate about whether the intervention scenario should have the user as the ‘main character’ with the content showing their future, or make use of testimonials from ex-smokers, showing their timeline of quitting, and the benefits they have experienced. The consensus supported having the user at the centre of the VR scenario.

*Yeah, I think to see case studies is a lot more… ‘cause it's real, isn't it? It's real people and their real stories… So, it makes it believable then, isn't it? Because it's true stories* **(53F, tobacco dependency advisor)**

*I think the inspirational, motivational stories and case studies are really, really, really helpful. But I think it's also really good to try and be able to somehow put yourself in those situations, because, you know, you can be motivated by what you're seeing from other people, but then you might think, ‘well, I can't do that because of this’ and put your barriers back up again. So, I think it would be good in some way to try and have like a timeline or showing how if you stop smoking for this number of days, you will get this much money like you said, or your lungs will be like this*. **(50F, stop smoking advisor)**

One suggestion was to use a gamification approach, where brief challenges would be completed and the ‘reward’ for completing that challenge would be a short clip showing a new benefit of quitting smoking.

*You would need to make it attractive and appealing on that front. I tell you, if you had an interactional aspect to it where people could actually fulfil a task during it and actually make it game like.* (**52M, clinical lead for tobacco dependency and mental health)**

*The VR thing, it would have to be like a game, to progress to the next level and things. And watch a case study in between, you know, like somebody who's not smoked for four days- how do they feel?* **(53F, tobacco dependency advisor)**

While experts in this study preferred communicating to their patients the benefits of quitting, they recognised that experiences of smoking are unique, and some smokers might respond more to content on the negative health consequences. Suggestions focused on using a decision tree to tailor the content shown to smokers. In conventional media, ‘choose your own adventure’ books or television programmes allow users to make decisions, which alter the next stage of the story. Consequently, different viewers receive a slightly different story. The final VR scenario shown to a specific user could depend on their demographics, smoking history or answers to questions posed within the scenario. This would also add an element of interactivity.

*I think you get games like that anyway, where you can… kind of like ‘choose your own adventure’ story books, when they came out. You could make a decision about where you go and change the ending if you do the game again* **(43F, stop smoking advisor)**

*Unless that's part of your screening beforehand, you sort of see what type of thing they are likely to respond to. So, then you lead them along a path.* **(49M, superintendent pharmacist)**

Regarding the technological execution, VR experts generally preferred the use of computer graphics and animation, compared to 360-degree filming. While 360-degree filming is less expensive, the use of animation allows for more customisation and interaction. For example, if showing alternative versions of the future, the user could create an avatar (meta- human) that resembles them, making the content feel more personally relevant.

*Whether it's interactive or not, make it animated because you could have someone customise an avatar and then they feel like the avatar resembles themselves. Either they customize the older version of them, or you build something that kind of makes it older.* **(27F, technical artist)**

*If you had an avatar that was personalised looking more towards you and it was more hopeful with all the positives, I think people would be much more accepting of that* **(37F, psychiatrist)**

One VR expert claimed that the use of computer animation did not need to be photorealistic for users to be immersed. Conversely, they thought that trying to use overly sophisticated animation could break immersion as most commercial and stand-alone headsets do not have the processing power to run that level of animation smoothly.

*One thing you might need to be aware of… is that sometimes people might think that because we're simulating the* *real world,* *we need to create something that's really highly photorealistic. But the reality is, if you try to do that,* *but then you put it in like a Quest 2 [headset] or something, it doesn't really run that* *well and you don’t have a very smooth experience. All those things can really break the immersion and the* *experience.* **(27F technical artist)**

Furthermore, in previous co-design studies with adult smokers, family members were suggested as supporting characters in the VR scenario, to increase the emotional salience of content showing the future.^26^ In this study, experts in smoking cessation agreed that this could be a useful tactic and corresponds to the BCT ‘inform about negative social consequences’. In healthcare experts’ previous interactions with patients, family members such as grandchildren had been cited as a reason to quit.

However, experts across groups thought this might be best utilised as a customisable option, depending on the user's background and relationship with their family. Regarding the technological execution, the use of NPCs (non-player characters) can increase the interactivity of a VR scenario. One of the experts in VR described her experiences with smokers using a cue-exposure therapy scenario that she had created.

*But, because they [NPCs] are talking to you, they are looking you in the eyes and they're interacting with you, people can find that highly immersive. And when they [the NPCs] were talking to them [the user], inviting you to smoke, they [the user] actually reply and they even give out their hands to be like, ‘Yeah, I want to grab that thing.’* **(27F, technical artist)**

Some participants suggested using artificial intelligence (AI), such as large language models and ChatGPT to inform the conversations between the user and NPCs, in contrast to pre-scripted dialogue. However, they cautioned that unless the AI was highly trained this could lead to unexpected results.

*So, people can talk with virtual agents, that are telling them something. So now, you know, the large language models are giving lots of freedom. In this case they can reply like they are humans.* **(48M, VR research associate)**

*We did a project where you hook a meta-human with chatGPT. Basically, when you [the user] are talking to the person [NPC], they are able to- because they are hooked with chatGPT- they are reacting to what you say. So, you're basically talking to a real human, but then they are kind of a bit dumb because chat GPT is a bit dumb in a way. Sometimes they keep on replying something that's irrelevant* **(27F, technical artist)**

Other technical features to increase immersion included spatial audio combined with noise-cancelling headphones to reduce distractions. Also, VR experts recommended using music to contribute to the atmosphere of the scenario.

#### Subtheme 1: unsuitable content – the potential problems with extreme fear appeals

Most smoking cessation experts were not enthusiastic about the second category of content suggested by adult smokers in the previous co-design study (horror stories using surreal images).^26^ They felt that an over-emphasis on scary images or graphic content could cause users to become fatalistic or defensive.

*I totally agree with all that and there needs to be a careful balance between fear and the general appeal because you don't want to trigger fatalism, you know, where someone abandons all hope* (**28F, tobacco dependency advisor)**

One healthcare expert who was also an ex-smoker explained,

*I think as well, if anybody sees a picture that they find offensive, they're not going to look at it, are they? So if you see any image that you find offensive to look at, you're not going to look at it in depth. You’re just going to brush past it really. I never really looked at- there was a photo on a packet of Lambert and Butler, one with somebody's throat, like with this growth of cancer or something else. And I once kind of glanced at it, but I never looked at it like fully, because I didn't want to look at that.* **(53F, tobacco dependency advisor)**

Also, some smoking cessation experts felt that rather than triggering anxiety about smoking, and consequently a quit attempt, such content would be more likely to be seen as entertainment and not taken seriously.

*Oh, so I don't know if there's going to be an element of the population that might actually find this entertaining as an image, and it might have the converse reaction, that they might think it's actually quite a fun image rather than actually driving them to make a behavioural change.* **(59F, Healthy living hub advisor)**

#### Theme 2: technical features – safe, inclusive and easy to use VR for a smoking population

Across all focus groups, experts highlighted how VR may not be suitable for every smoker. Healthcare experts felt that certain medical conditions would make the VR difficult to use or may cause harm. For example, the immersive quality of VR might be triggering for patients with a severe mental illness. Also, VR might not be suitable for those who are physically frail, or with visual and hearing impairments or those on specific medications. Experts suggested that practitioners should use a screening process to identify which smokers the VR would be appropriate for, to limit the chances of negative effects. Screening criteria could potentially exclude very old patients, those with hearing impairments, certain mental health conditions and those who are extremely unwell.

*It probably wouldn't be appropriate, especially within mental health, if patients are particularly, kind of, psychotic, then actually having something that's a virtual reality might actually just be off-putting, playing into their delusional experiences.* **(37F, psychiatrist)**

*For example, there is zyban as well, it's a stop smoking medication that lowers the threshold for seizures.* *Would they be able to do it?* *Things like that?* *Maybe consider.* **(43F, stop smoking advisor)**

*So, obviously like you're saying, it wouldn’t be ideal for ‘Betty’ who is 90, but I think if we did it on a screening basis then that's something that could be another resource that we could use.* (**29M, tobacco dependency advisor)**

Some experts across the focus groups felt that age should be part of the criteria used for screening, with older smokers screened out. The basis for this was that older smokers would not have the digital skills needed to use the VR equipment. Also, older patients would be more likely to have hearing and visual impairments.

*I think the challenge is that lots of people, they do not know how to use the virtual reality, and you might need to spend lots of time training the people, like how to use headsets, use the hand controller and it may only be suitable for young people. Like, older people might not easily accept that this is something that could help them.* (**24F, graduate student in VR)**

Additionally, some participants felt that older smokers would not be interested in using new technology. However, one healthcare expert disagreed and thought that older patients would be open to VR, if offered and feasible.

Another health and safety consideration was hygiene. Headsets cover the eyes and touch the nose, so would need to be disinfected between each use. Another suggestion to make VR easier was using hand tracking instead of controllers, as this would avoid the user needing to learn the function of each button. Also, having the VR scenario available in multiple languages would increase the inclusivity of the intervention.

*VR controllers can sometimes be a little bit challenging for people, like if someone is not much familiar with headsets, let's just say with the Quest, there are like four buttons and there are different triggers. So, people can often get confused with that. Also, when I was working with that rehabilitation thing, most of our patients were older, so for that, we used hand tracking*. (**25M, unity developer)**

*Obviously in [redacted, city], there's a lot of people who speak different languages. So, would it have multiple languages? Because there's so many times, I have to use our translation service to get an interpreter on the phone or face to face when I'm seeing a patient* **(32M, senior practitioner)**

Other suggestions to avoid unintended side effects included avoiding tasks within the VR scenario that include fast motion or running, as this would minimise cybersickness, also, avoiding flashing images in the scenario. The VR scenario should not be too long with too many tasks, as this could make the user physically tired and also result in cognitive overload. Finally, some headsets have external sensors that can detect the movement of external objects, which could prevent falls or tripping.

#### Theme 3: implementation – VR within the ‘ask, advise, act’ model in healthcare

Healthcare locations were the primary implementation suggestion, although there were some suggestions regarding using VR in occupational health.

*I was gonna suggest possibly the local surgery. It could be put there. I'm not sure were about. You know, in a private consulting room or in a pharmacy as well* **(70M, pharmacist)**

*I know in our trust for our diabetic retinopathy we have mobile treatment vans and stuff which have the equipment in the back of them…you could pull your trailer or your van into a market square or something, and do it ad hoc with people as well* (**52M, clinical lead for tobacco dependency and mental health)**

Regarding how VR could fit it in with current healthcare practices, one smoking cessation expert emphasised the 3As model of ‘ask, advise, act’. Concerning ‘ask’, some participants noted how within both primary and tertiary care it was difficult to identify the target population of smokers. Some experts also thought that certain locations were not suited to reaching smokers of a particular age group. This feeling was particularly strong among experts who felt VR was better suited to younger adults.

*The way I see it, a GP cannot accommodate, because young people don't go to the GP… They'd rather go to A and E [Accident and Emergency]. The young person will not literally walk into the GP because it's kind of like they're wasting their time. They just want something quick.* (**51F mental health student nurse)**

Some smoking cessation experts felt that some healthcare professionals did not often enquire about a patient's smoking status or were hesitant to offer advice if the patient indicated that they had no plans to quit.

*I also find as well that the question of ‘Do you smoke?’ on the admission is very often missed. So, our admitting doctors- clinicians -are not screening patients, so it's multiple missed opportunities* (**30F, tobacco cessation lead)**

*We get clinicians who are very anti any support once the person has indicated that they're going to smoke still. It can be quite tricky* (**52M, clinical lead for tobacco dependency and mental health)**

Sometimes, time was cited as a reason for a lack of enquiry. Other times, it was not knowing what to say.

*Maybe it's also a time thing again? ‘I've got all these customers I need to serve, I need to do this.’ So if they get into that conversation, they feel, ‘Oh my goodness, it can end up in a rabbit hole. And I need to do other things’. So, it's not knowing what to say and the timing.* (**40F pharmacist and health coach)**

*And I think we also have- I would say it's a lack of resources- usually, the community teams or the community will have a lot more input in terms of smoking cessation nurses and clinics, whilst [in] the inpatient wards we don't get as many people coming to kind of help us out* **(37F psychiatrist)**

Regarding, ‘advise’ the majority of smoking cessation experts were enthusiastic and thought the VR scenario could be a useful tool when advising patients to quit smoking. Theme 1 discusses the content that could be used to provide advice. In contrast, one smoking cessation expert questioned if using VR would save time and have added value compared to traditional videos.

*When I say resource, I don't necessarily mean money- although it probably is a big impact- because it's not just the cost of the VR headset. I think, thinking about the nature of the VR headset, where people have to actually put it on their heads, take it off and all of that, with public use, it could break easily because people can very easily drop one and also, they probably need to have someone there to kind of show them how to use it. So, then you're still using manpower to monitor that. So, it could take a bit longer than if it was a regular video because it takes time to adjust to it.* (**27F, tobacco dependency advisor)**

Finally, regarding ‘act’ most healthcare experts emphasised the need for follow-up action to provide support to the patient when their motivation is highest. Suggestions for follow-up support included booking an appointment with a stop-smoking counsellor, linking the VR intervention with a smoking cessation smartphone app and providing advice about pharmaceuticals. Healthcare professionals reported that often patients did not know the most optimal way to use nicotine replacement therapy (NRT) (dual use of patches for slow release and gum or lozenges for fast release, in the correct dose).

*I mean we're all using the model in general of ‘Ask, Advice, Act’. Sort of like we've identified the people who smoke. We've covered the ask side of it. The VR intervention may well be advising the person on the best thing they can do is to stop smoking and how they can do that. But then you need to include the act part. You can't just say, ‘OK, come on out and off you go. Thank you for coming’. You have to be able to act. You have to be able to follow that up otherwise the intervention itself is flawed.* **(52M, clinical lead for tobacco dependency and mental health)**

## Discussion

### Summary of findings

This study consisted of four focus groups with intersectoral experts in healthcare or VR. The goal was to refine the ideas proposed by adult smokers from a previous co-design study.^26^ In this current study, we developed three main themes and one subtheme on: VR content suggestions that build upon previous ideas by adding appropriate BCTs and technical features (theme 1); the potential unsuitability of previous ideas based on extreme fear appeals (subtheme 1) ; challenges and proposed solutions to make VR safe and inclusive (theme 2) and how to feasibly incorporate VR in healthcare settings, using the 3As model (theme 3).

In the previous focus group with adult smokers, two types of content were proposed. First, ‘coming of age’ stories, where the user would be transported to the future showing either the consequences of continued smoking or the benefits of having quit. Second, ‘horror stories’ were proposed, with a focus on graphic or surreal images. In this study, experts were sceptical about the ethicality and effectiveness of extreme fear appeals. Consequently, they preferred the first category of content. Suggested BCTs for supporting a ‘coming of age’ approach included ‘prompt comparative imagining of future outcomes’, ‘imagine reward’ and ‘inform about positive health consequences’.

Survey data suggest that most quit attempts in England are motivated by concerns over future or current health.^8^ Previous studies developing or testing VR for delivering smoking cessation messages have focused predominantly on the negative health effects of smoking and eliciting negative emotions. For example, the user being told they had received concerning information regarding a lung scan.^40^ Also, other VR scenarios involved watching healthy lungs decompose and ending up in hospital room or showing the economic implications, familial stress and ‘eventual sombre fate’ of smokers.^10,41^ In contrast, while experts in this study preferred more positive content, they suggested a ‘choose your own adventure’ scenario, where the types of futures and clips shown to participants would be dependent on their demographic characteristics or response options to questions posed within the scenario. This would have the benefit of making the scenario interactive, while also deviating from a one-size-fits-all approach.

There is some debate in the literature on which emotions elicited from smoking cessation media best predict quit attempts. Fear can motivate efforts to escape from the perceived threat, while sadness can lead to withdrawal, rumination and inaction.^42^ In contrast, hope is seen as a motivational force, which helps with perseverance when changing addictive behaviours.^42^ In a cross-sectional study of Australian smokers, potential exposure to hope or sadness-inducing campaigns was positively associated with thinking about quitting. However, it was exposure to campaigns evoking multiple negative emotions (fear, guilt, sadness) that predicted 30% of actual quit attempts.^43^ Conversely, in an English study analysing cessation campaigns, positive content resulted in more calls to quit lines.^5^ Ultimately, content aimed at persuading smokers to quit needs to strike the right emotional balance. A potential benefit of a ‘choose your own adventure’ scenario is that during evaluation, the content chosen by each user could be directly tracked and linked to outcome data.

While most experts in the study recommended having the user as the main character, three experts suggested having ex-smokers as the main character, as this could be inspirational. An analysis of mass media campaigns in England between 2004 and 2010 showed that only 17% of campaigns featured testimonials from real-life ex-smokers and their friends or relatives.^44^ Additionally, one VR scenario in the USA featured anecdotes from real-life smokers.^21^ However, most experts in this study felt that content focused on the user would feel more personally relevant. In VR, the user could be represented with an avatar. ‘Avatar embodiment’ refers to the users’ ability to process the properties of a virtual body in the same way as their physical body.^45^ Consequently, if something happens to the avatar (good or bad), the user feels as if it is happening to them.^45,46^ For example, an experiment used non-VR avatars (from Second Life) to test how the degree to which American university students emotionally bonded with their avatar affected their alcohol-related behavioural intentions when the avatar experienced a negative consequence (car crash after intoxicated driving).^46^ The results showed that being able to customise the appearance of the avatar (facial features, clothing, skin tone, musculature) compared to a pre-designed avatar predicted emotional closeness. Additionally, a higher degree of emotional closeness to the avatar was associated with positive intentions to change behaviour. However, the ability to customise the avatar did not directly affect behavioural intention.^46^

These results suggest that the use of customisable avatars could be an important mediating (rather than direct) factor in the behaviour-change pathway. In the context of developing a smoking cessation VR scenario, using customisable avatars may make the content of the scenario feel more directly applicable to the user, compared to watching content based on other people. Practically, we suggest that having a small (rather than exhaustive) menu of features to pick from may be a useful way to incorporate customisable avatars without making the set-up process for the VR scenario too long, especially if used in a time-sensitive setting such as a healthcare location. One expert in the VR focus group recommended the platform ‘Ready Player Me’ for custom avatar integration.^47^

Previous VR scenarios have been predominantly passive, with little opportunity for interaction or customisation.^21,40^ These scenarios generally relied on smartphone-based VR, which provides a 360-degree view, but the user cannot pick up objects.^48^ In this study, experts in VR recommended features to make the VR scenario more interactive and engaging. As discussed previously, some of these recommendations included being able to alter the story content and using customisable avatars. Another suggestion was using AI to inform any dialogue spoken by NPCs. In gaming, AI has been used to develop behaviours for NPCs and generate dynamic dialogues between the user and NPCs.^49^ In particular, generative AI and large language models are trained using extensive datasets and can produce texts that are almost indistinguishable from human-authored scripts.^50^ This can provide a tailored storytelling experience, especially when combined with text-to-speech functions.^50^ However, one expert in this study noted that unless the AI was carefully reviewed and tightly constrained, it can be unpredictable, which poses a significant drawback.

Experts in VR generally recommended using computer graphics over 360-degree panorama filming. Although experts reported that 360-degree filming is less expensive and more achievable, they felt it was limited in terms of customisation and interactivity. Smoking cessation experts implicitly endorsed computer graphics with references to avatars and customised animation, although they were not asked about preferences directly. An experiment comparing a filmed panorama to a computer graphic scenario found that users experienced similar levels of presence.^51^ However, this study accounted for the limitations of 360 filming by keeping the level of tracking and interactivity the same in both versions. Additionally, VR experts in this study suggested using spatial audio to increase the user's sense of presence, and music to evoke emotions. Music and sound effects have been used for a similar purpose in previous smoking cessation VR scenarios, such as Caponnetto and colleagues’ use of ‘Requiem for a Dream’ by Aronofsky and ‘tolling of bells’ sound effects to represent ‘virtual death’.^10^ Finally, other suggestions from a similar focus group study with experts in health and VR were being able to customise the virtual background, and olfaction (the smell of cigarettes contrasted with the smell of fresh air).^41^

In VR, there needs to be a balance between immersive features, safety and inclusivity. In this study, experts in healthcare recommended a screening process to screen out individuals who cannot safely or effectively use VR, such as those with severe illnesses, psychiatric conditions and patients on medications that increase the risk of seizures. However, digital exclusion is when certain subgroups are unable to benefit from technology.^52^ Therefore, from a health equity perspective, the VR intervention should be as inclusive as possible. In this study, suggestions included making the VR available in multiple languages. Also, using external sensors to avoid the risk of tripping, avoiding fast motion within the virtual environment to limit cybersickness/motion sickness and limiting the number of tasks and the length of the scenario to avoid tiredness and cognitive overload. Smoking cessation experts also noted the importance of hygiene and making sure the VR headset was disinfected between uses. In a similar focus group study for smoking cessation, with experts in VR, an additional suggestion was to program the VR to be tolerant of input errors. When users are frustrated with technical difficulties this can also break immersion.^41^ There was some disagreement among experts concerning whether older smokers would be interested in using VR. We have previously reported on a cross-sectional representative survey of British smokers that interest in VR was similar across all age groups, except those over 65, where interest was significantly lower (OR: 0.29, 95% CI: 0.15–0.57).^53^ In a scoping review of VR design considerations for older adults, recommendations included creating seated experiences, having navigation aids and demonstrations beforehand, large fonts and objects, and a simple, intuitive user interface.^54^

The last theme concerned implementation challenges. Similar to our previous focus group study with adult smokers, healthcare locations such as pharmacies, clinics and GPs were the main locations recommended.^26^ While, the 3As model has been successful in increasing quit rates,^38^ experts in this study identified challenges and recommendations when adding a VR element. Regarding ‘ask’, experts noted that identifying patients who smoked was often a missed opportunity and cited time and not knowing what to say as the main barriers. Similarly, previous qualitative studies indicate that most doctors do not broach the subject of smoking unless the patient initiated the discussion, or they could link smoking to the condition that brought the patient into the office.^55^ Also, some GPs feel that they lack the confidence, time and skills needed to effectively engage with smokers who have shown no interest in quitting, beyond giving general advice.^56,57^ While VR may be a useful ‘icebreaker’ or conduit for providing brief advice, experts in this study noted that it may not solve the issue of time constraints if the VR headset has to be monitored, set up and cleaned by a healthcare professional. A systematic review of the barriers and facilitators of the implementation of VR in healthcare identified having a private room for the VR, and technical training for staff as key facilitators.^58^

Regarding ‘act’, healthcare experts in this study highlighted the importance of follow-up action after the VR, similar to our previous focus group, where adult smokers questioned if a VR scenario was a long-term solution.^26^ Smoking cessation experts highlighted the importance of long-term behavioural and pharmacological support, and some experts recommended linking the VR to a cessation smartphone app. We suggest that these follow-up (act) steps could be incorporated into the VR scenario itself (e.g., an option to enter your email, to receive a link to download a smoking cessation smartphone app) or be delivered by the healthcare professional (a referral to behavioural support).

### Design considerations moving forward

Based on the results of this study, Table 3 outlines our guiding principles (a heuristic used in the PBA) for a minimal viable product which could provide proof of concept. However, delivery technology is rapidly changing with newer, more advanced, and more affordable headsets being released.^59^ The pace of technological change in industry outstrips the much longer timeframes needed to create evidence-based health technologies in academia and behavioural science, using frameworks such as the PBA.^23,24,60^ Therefore, Table 3 also includes considerations we recommend based on the results of this study, which may not be essential for a minimal viable product in the UK.

**Table 3.** Guiding principles for a minimal viable product based on the results of the present study.

User context	Design objectives	Intervention technical features and BCTs
Smokers may become defensive or inattentive if extreme or graphic imagery is used. They may also become defensive if judgemental language is used.	An approach which is empathetic, which triggers positive action, rather than inaction or rumination.	Empathic communication style Non-judgmental communication style Minimise ‘increase salience of consequences’ BCT
Some smokers may respond better to the benefits of quitting while some may respond more to the consequences of continuing to smoke.	Provide a tailored VR experience.	The ability of the user to choose which clips of future outcomes are shown. Inform about negative health consequences BCT. Inform about positive health consequences BCT. Imagine reward BCT. Prompt comparative imagining of future outcomes BCT. Inform about negative social consequences BCT. Adopt changed self-identity BCT. Affirm valued self-identity BCT.
Smokers may discount the messages of traditional smoking cessation as not applicable to them.	Embodied content which feels personally relevant to the user.	The ability to customise an avatar, to resemble the user.
Smokers may find immersive content more engaging and compelling than traditional media.	User audio features to increase the users’ sense of presence within the virtual environment.	Spatial audio Sound effects and music Noise cancelling headphones
Older smokers or those with limited digital skills may struggle to use VR.	An experience which is easy to use, with minimal complications.	The scenario will use standard controllers. A user demonstration will be included before the main scenario. Hand tracking instead of controllers.^a^
Smokers may be at risk of cognitive overload, cybersickness or fatigue while using VR.	Content, which is long enough to achieve its aims, but contained to avoid unintended harms.	Time limit on the scenario. Limited physical tasks to avoid fatigue. A seated experience. Avoid fast graphics and flashing images to minimise cybersickness.
Smokers require sustained motivation for a successful quit attempt. Smokers are usually unaware of the most optimal method of using NRT	After the VR scenario smokers must be signposted towards additional support.	An option for the user to enter their email to provide with additional information and links in writing. Encourage pharmacological support BCT.
For some smokers, English is not their first language or do not speak English.	An experience that can be understood by non-English speakers.	Have the dialogue available in multiple common languages.^a^

a Desirable feature, but may not be feasible for an initial prototype.

### Limitations

The main strength of this study is the diverse sample of health professionals, from various disciplines, working in different health settings. Often, healthcare professionals are not included in the design of health-related technology in a meaningful way.^20^ Also, the VR experts were able to provide a unique perspective regarding technical considerations for the intervention. However, only one focus group included experts on VR, due to recruitment and time constraints. We may not have achieved theoretical saturation on VR-specific technical considerations. Additional focus groups with the VR experts may have been helpful to discuss the cost implications of the technical suggestions from previous groups.

A disadvantage of online focus groups is that they may not be suitable for older individuals, or those with unstable internet connection.^32^ In this study, participants were either VR or healthcare professionals, who most likely use technology as part of their work practice. Only one participant in group 3 faced significant technical issues which impacted their ability to participate in the discussion.

## Conclusion

In conclusion, this focus group study recruited 30 experts in healthcare and VR to refine the user-generated ideas from a previous study with adult smokers. Participants recommended VR content that showed or compared different versions of the future which maps on to behaviour techniques such as ‘comparative imagining of future outcome’, ‘imaginary reward’ and ‘information about positive health consequences’. Experts in VR recommended the use of computer graphics to enable customisable avatars. Regarding implementation, while healthcare locations were preferred, there were some concerns about the time requirements needed to set up VR in a clinical space. A screening process might be necessary to avoid conferring harm on certain subgroups of the population.

This study is nested within a larger project aimed at developing a VR scenario to prompt and encourage smokers to quit. Moving forward, the design and content considerations from this study will inform the development of a prototype, which will then be further refined and tested using ‘think aloud’ methodologies with the target population. While a single VR intervention may not satisfy all members of the target population, its development represents a step forward in novel smoking cessation methods.

## Supplemental Material

sj-pdf-1-dhj-10.1177_20552076251330510 - Supplemental material for Identifying behaviour change techniques, technical features and implementation options for a virtual reality intervention to motivate adult smokers to quit: A focus group study with healthcare and virtual reality experts Supplemental material, sj-pdf-1-dhj-10.1177_20552076251330510 for Identifying behaviour change techniques, technical features and implementation options for a virtual reality intervention to motivate adult smokers to quit: A focus group study with healthcare and virtual reality experts by Tosan Okpako, Corinna Leppin, Alessandro Chincotta, Dimitra Kale, Olga Perski and Jamie Brown in DIGITAL HEALTH

sj-docx-2-dhj-10.1177_20552076251330510 - Supplemental material for Identifying behaviour change techniques, technical features and implementation options for a virtual reality intervention to motivate adult smokers to quit: A focus group study with healthcare and virtual reality experts Supplemental material, sj-docx-2-dhj-10.1177_20552076251330510 for Identifying behaviour change techniques, technical features and implementation options for a virtual reality intervention to motivate adult smokers to quit: A focus group study with healthcare and virtual reality experts by Tosan Okpako, Corinna Leppin, Alessandro Chincotta, Dimitra Kale, Olga Perski and Jamie Brown in DIGITAL HEALTH
