# Supplementary material for: Identifying behaviour change techniques, technical features and implementation options for a virtual reality intervention to motivate adult smokers to quit: A focus group study with healthcare and virtual reality experts
Source: Digit Health. 2025 Mar 28;11:20552076251330510. doi: 10.1177/20552076251330510 (PMC11951901; doi:10.1177/20552076251330510)
Supplement: sj-docx-2-dhj-10.1177_20552076251330510 - Supplemental material for Identifying behaviour change techniques, technical features and implementation options for a virtual reality intervention to motivate adult smokers to quit: A focus group study with healthcare and virtual reality experts [file sj-docx-2-dhj-10.1177_20552076251330510.docx]

**Supplementary Material**

**Supplementary material 2**

**Table S1. Baseline Screening Questionnaire for Intersectoral stakeholders**

| **What is your age (in years)?** | 0-120 |
| --- | --- |
| **Do you have work or research experience, in computer science, VR development, digital health or theory-based smoking cessation interventions (e.g., GP, stop smoking advisor, academia)?** | 1. No 2. Yes |
| **Are you willing to attend a focus group session online?** | 1. No 2. Yes |

| **What is your name?** | Free text |
| --- | --- |
| **What is your email address?** | Free text |
| **What is your mobile phone number?** | Free text |
| **Which of the following describes how you think about yourself?** | 1. Male 2. Female 3. In another way 4. Prefer not to say |
| **What is your ethnic group?** | 1. Any Asian or Asian British background 2. Any Black, Black British, Caribbean, or African background 3. Any White background 4. Mixed or multiple ethnic groups (e.g., White and Black African or White and Asian) 5. Other ethnic group (e.g. Arab) |
| **What is your work or research field? Select all that apply** | 1. Virtual reality/ computer science- industry 2. Virtual reality/ Computer Science- academia/ research 3. Digital health- industry 4. Digital health- academia/research 5. Health- academia/ research (e.g., epidemiology, behavioural science, psychology) 6. Healthcare (e.g., GP, pharmacist, stop-smoking counsellor) |
| **What is your current job title?** | Free text |
| **What are you preferred dates to join a one-off focus group, online? (select all that apply)** | 1. TBD 2. TBD 3. TBD 4. TBD 5. TBD |
| **Would you like to receive a copy of the final study results when ready?** | 1. No 2. Yes |

**Supplementary material 3**

**Table S2. Question route for smoking cessation experts**

| *Opening question* | - Introduce moderator (T.O.) and co-facilitator(s) - Explain the aim of the focus group. Including an explanation of VR. - Discuss confidentiality and ground rules for discussion. - Explain what VR is - Can we go around and introduce ourselves, giving our first name and describe in one or two sentence our professional background and how it relates to smoking? |
| --- | --- |
| **Begin audio recording** | |
| *Introductory question* | 1. In your professional and research experience, what would you say are the main barriers and facilitators to advising adults to quit smoking? 2. Has anyone here ever used VR before? What do you know about it? |
| **Present slides explaining what VR is in sufficient detail.** | |
| *Transition questions* | 1. Can you think of any settings outside of people’s homes where a smoking cessation VR app could feasibly be used or implemented?    - If no suggestions give prompts (e.g. GP office) 2. What do you think would be the biggest challenges or benefits of incorporating VR into these settings?    - Prompt: (for healthcare professionals) Would you be for or against incorporating VR into your own work practice? Why or why not? 3. How could any potential challenges be mitigated? |
| **Present a slideshow summarising the two broad categories of content suggested by adult smokers (previous focus group study).** | |
| *Key questions* | 1. In your professional experience, to what extent do you feel like this type of content would appeal to adult smokers?    - Prompt: any strengths or weaknesses?    - Prompt: (specifically for the horror content) Do you think it would be ethical to show graphic content in this style? 2. What behavioural support, strategies or tips do you think could be incorporated into these ideas that could strengthen their potential to increase motivation to quit smoking cigarettes?   **Repeat questions 6 and 7 for the second category of content.** |
| *Closing questions* | - Does anyone have anything else they would like to share? - Does anyone have any questions? - Thank participants for their time. |

**Table S3- Question route for virtual reality experts**

| *Opening question* | - Can we go around and introduce ourselves, giving our first name and professional background? |
| --- | --- |
| **Begin audio recording** | |
| *Introductory question* | 1. Generally speaking, what would you all say are the integral features of VR that make it unique compared to other forms of technology and media delivery? 2. Does anyone have any experience using or developing VR for health behaviours? |
| *Transition questions* | 1. Can you think of any settings outside of people’s homes where a smoking cessation or health focused VR app could feasibly be used or implemented?    - If no suggestions give prompts (e.g. GP office) 2. What do you think would be the biggest challenges or benefits of incorporating VR into these settings? 3. How could any potential challenges be mitigated? |
| **Present a slideshow summarising the two broad categories of content suggested by adult smokers (previous focus group study). Questions 5-8 will be repeated for each broad content category.** | |
| *Key questions* | 1. Do you think these ideas would be better suited to animated graphics or motion picture (real-life) filming- why or why not? 2. Does anyone have any suggestions about where user interaction could be incorporated and how it can be incorporated? 3. Does anyone have any suggestions regarding opportunities to increase the immersive properties of these ideas?    - Prompt: If there are no suggestions specific to the smoking content, ask what general strategies are used to increase immersion in VR. 4. Is there a potential for customisation or tailoring? If so, in what ways?    - Prompt: in no response, give suggestions to trigger conversation (e.g., choosing an avatar or the ability to make choices that alter the direction of the content)?    - Prompt: How feasible would this [suggestion] be? 5. Are there any specific features of VR that increase the likelihood of unintended harm?    - Prompt: for example, becoming emotionally overwhelmed, cybersickness or dizziness    - Prompt: How could these be mitigated or minimised? |
| *Closing questions* | - Does anyone have anything else they would like to share? - Does anyone have any questions? - Thank participants for their time. |

**Supplementary 4**

**Research team and reflexivity**

This study forms a part of TO’s PhD thesis. TO is a female PhD student with an MSc in Public Health. TO does not smoke cigarettes or use any other type of nicotine products, so approached the topic of smoking cessation from an academic perspective, rather than lived experience. Also, TO is more familiar with the professional language and practices of health and behavioural sciences in contrast to the practices of computer science. This may have influenced the data collection and analysis process in terms of which expert responses were probed further and which responses appeared novel.

CL is also a PhD student, while AC is a master’s student in Health Psychology. TO and CL have previous experience conducting focus groups for digital health studies. The study participants (experts) did not know TO, CL or AC. There was minimal contact before the focus groups except for a reminder email. To obtain informed consent, we provided experts with some details of the research team and project in the participant information sheet. While experts were aware of the project’s main aims, we emphasised during the focus groups that all perspectives were welcome, to minimise social desirability bias.
